# Protective CD8^+^ T cell memory without help

**DOI:** 10.18632/oncotarget.5449

**Published:** 2015-09-01

**Authors:** Min Fang, Luis J. Sigal

**Affiliations:** Institute of Microbiology, Chinese Academy of Sciences, Beijing, P. R. China; Fox Chase Cancer Center, Philadelphia, PA, USA

**Keywords:** Immunology and Microbiology Section, Immune response, Immunity, CD8 T cell memory, CD4 T cell help

CD8 T cells are a key component of the host adaptive immune responses that helps to eradicate invading virus and other cell-associated pathogens. The CD8 T cell responses to an acute infection consist of three well defined phases: naïve pathogen-specific T cells (CD8_N_) become activated and expand resulting in large numbers of effector cells (CD8_E_); the contraction of these CD8_E_ into memory cells (CD8_M_) once the infection is cleared; and the long-term maintenance of these CD8_M_. If a secondary infection occurs, the CD8_M_ mount more vigorous and faster responses than CD8_N_, which help to rapidly and efficiently control the infection. The prolonged maintenance of this pool of antigen-specific CD8_M_ can help protect from certain infections. Hence, one of the goals of vaccination is to generate CD8_M_.

CD4 T cell help (T_H_) is essential for priming CD8 T cell responses to cell-associated, non-inflammatory antigens while being dispensable for responses generated to a variety of infectious pathogens. In several infectious models, T_H_ is critical for the conditioning and/or maintenance of the CD8_M_ pool and/or their secondary expansion and differentiation into secondary effectors.

VACV is an orthopoxvirus (OPV) that was used as the vaccine that eliminated human smallpox, a highly lethal disease caused by the human-specific OPV variola virus (VARV). VACV is regarded as the golden standard of a highly effective vaccine. In addition to preventing smallpox, VACV is also effective as a vaccine against lethal mousepox, a disease caused by the mouse-specific OPV ectromelia virus (ECTV). We previously showed that in addition to antibodies, CD8_M_ induced by VACV immunization can fully protect susceptible mice from lethal mousepox [1], suggesting that the establishment of a CD8_M_ pool is one of the mechanisms whereby the smallpox vaccine protects from pathogenic OPVs. However, during the course of VACV infection or immunization, the role of T_H_ for the generation, maintenance and recall responses of the anti-VACV CD8_M_ remained controversial [2-6]. A possible explanation for these discrepancies may lie in the replicative capacity of the VACV strain used in different studies. Using a non attenuated VACV strain WR as the vaccine and ECTV as the pathogen, and by measuring polyclonal rather than transgenic CD8 T cells responses, we have recently shown that anti-VACV CD8_M_ generated in the absence of T_H_ that expand and differentiate into CD8_E_ are as effective as helped CD8_M_ in their ability to protect from lethal ECTV infection [7].

Consistent with some previous research, we found that wild type B6 mice and MHC-II-deficient mice (MHC-II^0/0^), which lack MHC-II restricted T_H_, mounted similar CD8 T cell responses during the acute phase of VACV infection (i.e. 7 days post immunization), indicating that optimal primary CD8 T cell responses to VACV are T_H_ independent. After virus clearance, the frequency of CD8_M_ specific for the VACV immunodominant determinant TSYKFESV (also an immunodominant determinant of ECTV) declined faster in MHC-II^0/0^ mice. However, most of the activation and memory markers were similar between the TSYKFESV-specific CD8_M_ from wild type and MHC-II^0/0^ mice. Moreover, the unhelped CD8_M_ expanded and generated secondary CD8_M_ when maintained and boosted in the MHC-II deficient environment, and most of the activation and memory markers between the TSYKFESV-specific secondary CD8_M_ from wild type and MHC-II^0/0^ mice were similar.

The ultimate goal of CD8_M_ is protecting from disease. To test the protective potential of the unhelped CD8_M_, we transferred secondary CD8_M_ from wild type and MHC-II^0/0^ mice into B6.D2-(D6Mit149-D6Mit15) LusJ (B6.D2-D6) mice, a B6 congenic mouse strain that is susceptible to mousepox. Importantly, when adjusted to contain similar numbers of TSYKFESV-specific CD8_M_, the unhelped CD8_M_ protected B6.D2.D6 mice as efficiently as helped CD8_M_. Transferring as few as 4.5×10^4^ helped or unhelped TSYKFESV-specific CD8_M_ significantly reduced the virus loads to similar lower levels and fully protected B6.D2-D6 mice from death. Thus, polyclonal anti-VACV CD8_M_ generated in the absence or in the presence of T_H_ are similarly potent at protecting mice from lethal ECTV infection on a per cell basis.

**Figure 1:**
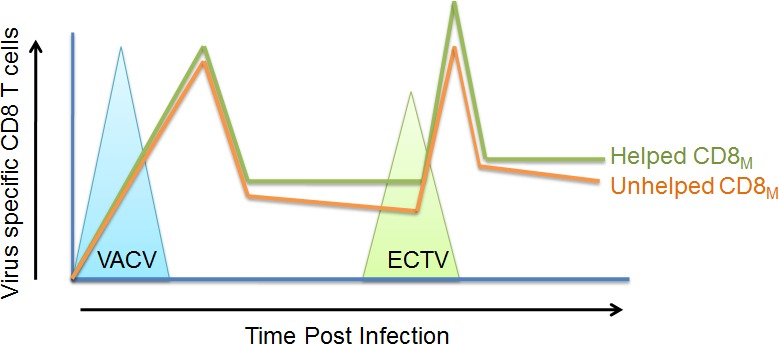
Conditioning and maintenance of anti-VACV CD8_M_ and their protective capability to ECTV infection can develop without T_H_ The primary CD8 T cell responses to VACV were similar between wild type B6 mice and MHC-II^0/0^ mice. Functional CD8_M_ were maintained in MHC-II^0/0^ mice even though at lower frequency. When cell numbers are adjusted, the unhelped CD8_M_ from MHC-II^0/0^ mice were similarly potent at protecting mice from lethal ECTV infection as the helped CD8_M_ from wild type mice.

Our results do not necessarily dispute that T_H_ contribute to optimal maintenance of CD8_M_ as the CD8_M_ declined faster in MHC-II^0/0^ mice than that in WT mice. Yet, it is possible that this faster decline was due to the general poorer health of MHC-II^0/0^ mice, which are immunodeficent. Nevertheless, our work clearly shows that T_H_ is not essential for the establishment of functional CD8_M_ or to confer CD8_M_ the capacity to protect from a lethal infection (Figure 1). Because VACV is used as a vaccine in humans, our results may help us to understand how this vaccine induces protective immunity in people.
